# Uncoupling associations of risk alleles with endophenotypes and phenotypes: insights from the *ApoB* locus and heart‐related traits

**DOI:** 10.1111/acel.12526

**Published:** 2016-09-28

**Authors:** Alexander M. Kulminski, Yelena Kernogitski, Irina Culminskaya, Yury Loika, Konstantin G. Arbeev, Olivia Bagley, Matt Duan, Liubov Arbeeva, Svetlana V. Ukraintseva, Deqing Wu, Eric Stallard, Anatoliy I. Yashin

**Affiliations:** ^1^ Biodemography of Aging Research UnitSocial Science Research InstituteDuke UniversityDurhamNC27708‐0408USA

**Keywords:** aging, *ApoB* polymorphism, endophenotypes, healthspan, lifespan, trade‐offs

## Abstract

Traditionally, genomewide association studies (GWAS) have emphasized the benefits of large samples in the analyses of age‐related traits rather than their specific properties. We adopted a realistic concept of genetic susceptibility to inherently heterogeneous, age‐related traits driven by the elusive role of evolution in their properties. We analyzed in detail the associations of rs693 and rs562338 polymorphisms representing the *Apolipoprotein B* locus with endophenotypes (total cholesterol [TC] and high‐density lipoprotein cholesterol) and phenotypes (myocardial infarction [MI] and survival) in four large‐scale studies, which include 20 748 individuals with 2357 MI events. We showed that a strong, robust predisposition of rs693 and rs562338 to TC (β = 0.72, *P* = 7.7 × 10^−30^ for rs693 and β = −1.08, *P* = 9.8 × 10^−42^ for rs562338) is not translated into a predisposition to MI and survival. The rs693_A allele influences risks of MI and mortality after MI additively with lipids. This allele shows antagonistic effects—protecting against MI risks (β = −0.18, *P* = 1.1 × 10^−5^) or increasing MI risks (β = 0.15, *P* = 2.8 × 10^−3^) and mortality after MI, in different populations. Paradoxically, increased TC concentrations can be protective against MI for the rs693_A allele carriers. Our results uncouple the influences of the same alleles on endophenotypes and phenotypes despite potential causal relationships among the latter. Our strategy reveals virtually genomewide significance for the associations of rs693 with MI (*P* = 5.5 × 10^−8^) that is contrasted with a weak estimate following the traditional, sample‐size‐centered GWAS strategy (*P* = 0.16) in the same sample. These results caution against the use of the traditional GWAS strategy for gaining profound insights into genetic predisposition to healthspan and lifespan.

## Introduction

Technological breakthroughs in genomewide genotyping raised enthusiasm for advancing the progress in discovering genes influencing various health traits. Advances in the field are promising for strengthening strategies for extending healthspan and lifespan (Franceschi *et al*., 2007; Sierra *et al*., 2008; Bloss *et al*., 2011). To address this problem, one necessarily faces the need to deal with genetic influences on age‐related traits, that is, on traits which are characteristic of the elderly people in modern societies. They are a special case of complex polygenic phenotypes, which have three important complications.

First, age‐related traits are a relatively new *widespread* phenomenon in human history. This is because, for example, in 1840 the world record of mean lifespan for women was approximately 45 years (Oeppen & Vaupel, 2002), implying that about half of the population did not survive to older ages where the incidence of age‐related health traits sharply increases. Second, they are characteristic of the postreproductive period where selection pressure is not as strong as at the reproductive period, and therefore, the role of evolution in these traits is elusive (Vijg & Suh, 2005; Crespi *et al*., 2010; Kulminski, 2013; Corella & Ordovas, 2014). Third, these traits appear in late life whereas genes are transmitted from parents at conception, that is, these events are separated by a large portion of the individuals’ life.

Currently prevailing genomewide association studies (GWAS) emphasize ‘the benefits of the large sample sizes achievable through collaboration’ (Willer *et al*., 2013), rather than specific properties of complex traits and follow the same strategy regardless of whether the analyses are focused on traits directly affecting fitness (Yang *et al*., 2010) or age‐related traits which do not directly affect fitness (Ikram *et al*., 2009).

It is often argued that GWAS of age‐related traits (phenotypes) can be improved by focusing on their precursors (called endophenotypes) (Willer *et al*., 2013). This strategy can be beneficial because endophenotypes can be characteristic for reproductive age and be under direct pressure of natural selection. Accordingly, one could expect evolutionarily established molecular mechanisms regulating endophenotypes that presumably should be translated into genetic predisposition to the related downstream phenotypes (Willer *et al*., 2008; Teslovich *et al*., 2010).

The problem, however, is that (i) endophenotypes have not been selected against or in favor of their pathological dysregulation causing age‐related traits (Crespi, 2010), and (ii) genes involved in regulation of normal function of endophenotypes were selected in principally different conditions than those in modern societies (Vijg & Suh, 2005; Nesse *et al*., 2012). Accordingly, one has the same evolutionary concerns about mechanisms of genetic regulation for endophenotypes as for phenotypes.

As a result, one should expect complex mechanisms linking genes with endophenotype and phenotypes. Then, a more realistic concept of genetic predisposition to endophenotype and phenotypes should adopt the complexity in gene actions rather than just rely on the existence of ‘a true effect’ (Button *et al*., 2013) with wide norm of reaction (Dobzhansky, 1973). This implies that it is not necessary that genetic effects on endophenotype and phenotypes should be the same in different populations (of even the same ancestry) and that genetic predisposition to endophenotypes may not necessarily be translated into genetic predisposition to downstream phenotypes.

We address the problem by considering in detail the associations of rs693 and rs562338 polymorphisms, representing the *apolipoprotein B* (*ApoB*) locus, with endophenotypes (total cholesterol [TC] and high‐density lipoprotein cholesterol [HDL‐C]) and phenotypes (myocardial infarction [MI] and survival) in four studies, that is, the Framingham Heart Study (FHS), the Atherosclerosis Risk in Communities (ARIC) Study, the Multi‐Ethnic Study of Atherosclerosis (MESA), and the Cardiovascular Health Study (CHS).

## Results

Basic characteristics of the ARIC, MESA, FHS, and CHS relevant to our analyses are given in Table 1 (see Section ‘Experimental procedures’, Subsection ‘Data’). Table S1 (Supporting information) also provides information on participants of each FHS cohort separately, that is, the FHS original (FHS), offspring (FHSO), and the 3rd generation (3rd Gen) cohorts.

**Table 1:** Basic characteristics of the genotyped participants of the selected studies

Factor	rs693			rs562338		
	GG	GA	AA	GG	GA	AA
Atherosclerosis Risk in Communities (ARIC) Study						
* N* (%^a^)	2378 (24.8)	4755 (49.6)	2459 (25.6)	6417 (66.8)	2897 (30.2)	291 (3)
Age, mean (SD), years	54.3 (5.6)	54.3 (5.7)	54.3 (5.7)	54.4 (5.7)	54.2 (5.7)	54.2 (5.8)
LS, mean (SD), years	69.8 (5.8)	69.9 (5.9)	69.9 (5.8)	69.9 (5.9)	69.8 (5.8)	69.6 (6.1)
TC, mean (SD), mg dL^−1^	210.1 (40)	215.6 (40.7)	218.6 (41.3)	217.5 (40.8)	210.2 (40.5)	208.2 (39.9)
HDL‐C, mean (SD), mg dL^−1^	51 (16.7)	50.4 (16.8)	50.2 (16.4)	50.5 (16.6)	50.6 (16.8)	49.3 (17.2)
MI, yes (%^b^)	195 (8.2)	395 (8.3)	176 (7.2)	524 (8.2)	227 (7.8)	18 (6.2)
Death, yes (%^b^)	373 (15.7)	701 (14.7)	353 (14.4)	945 (14.7)	436 (15.1)	45 (15.5)
Framingham Heart Study (FHS)						
* N* (%^a^)	2232 (26.3)	4045 (47.7)	2198 (25.9)	5403 (63.9)	2645 (31.3)	401 (4.7)
Age, mean (SD), years	37.8 (9.3)	37.8 (9.3)	37.7 (9.4)	37.7 (9.4)	37.8 (9.1)	37.9 (9.2)
LS, mean (SD), years	63.1 (17.1)	63.6 (17.5)	63.1 (17.4)	63.3 (17.4)	63.2 (17.1)	62 (17.2)
TC, mean (SD), mg dL^−1^ c	192.9 (40.7)	197.5 (39.8)	200.7 (42.5)	199 (41.2)	194.1 (40.2)	189.5 (36.5)
HDL‐C, mean (SD), mg dL^−1^ c	52.8 (15.2)	52.6 (15.4)	51.6 (15.7)	52.2 (15.4)	52.8 (15.5)	51.7 (14.9)
MI, yes (%^b^)	190 (8.5)	337 (8.3)	162 (7.4)	436 (8.1)	206 (7.8)	37 (9.2)
Death, yes (%^b^)	433 (19.4)	885 (21.9)	460 (20.9)	1149 (21.3)	536 (20.3)	71 (17.7)
Multi‐Ethnic Study of Atherosclerosis (MESA)						
* N* (%^a^)	729 (27.2)	1359 (50.6)	597 (22.2)	1691 (63.1)	875 (32.6)	114 (4.3)
Age, mean (SD), years	63.4 (10)	62.4 (10.2)	62.6 (10.1)	62.7 (10.1)	62.8 (10.2)	63.2 (9.9)
LS, mean (SD), years	71.4 (10)	70.5 (10.2)	70.8 (10.1)	70.7 (10.1)	70.9 (10.2)	71.6 (10.2)
TC, mean (SD), mg dL^−1^	193.6 (36.2)	194.5 (34.9)	201 (35.4)	197.2 (35.7)	194 (34.9)	185.9 (35.2)
HDL‐C, mean (SD), mg dL^−1^	52.9 (16)	52.3 (15.4)	52.2 (16.4)	52.6 (16.1)	52.4 (15.3)	51.1 (14.2)
MI, yes (%^b^)	18 (2.7)	36 (2.8)	22 (3.9)	46 (2.9)	28 (3.4)	1 (1)
Death, yes (%^b^)	54 (8.1)	93 (7.2)	29 (5.1)	98 (6.1)	70 (8.6)	8 (7.8)
Cardiovascular Health Study (CHS)						
* N* (%^a^)	1176 (26.6)	2182 (49.4)	1062 (24.0)	2834 (64.0)	1430 (32.3)	164 (3.7)
Age, mean (SD), years	72.5 (5.4)	72.9 (5.6)	72.8 (5.7)	72.9 (5.6)	72.6 (5.6)	73.1 (6.2)
LS, mean (SD), years	83.5 (5.2)	83.4 (5.4)	83.2 (5.5)	83.4 (5.4)	83.4 (5.3)	83.5 (5.3)
TC, mean (SD), mg dL^−1^	210.2 (38.8)	210.3 (39.5)	215.5 (39.2)	213.8 (40.1)	207.9 (37.9)	205.5 (35.3)
HDL‐C, mean (SD), mg dL^−1^	53.1 (15.7)	53.8 (15.8)	53.2 (15.3)	53.4 (15.8)	53.6 (15.3)	53.7 (17.1)
MI, yes (%^b^)	225 (19.1)	504 (23.1)	246 (23.2)	623 (22.0)	326 (22.8)	26 (15.9)
Death, yes (%^b^)	572 (48.6)	1129 (51.7)	549 (51.7)	1453 (53.2)	713 (49.9)	88 (53.7)

*N* denotes sample size.

Mean age is given at baselines.

LS denotes lifespan, that is, age at death or the end of follow‐up.

TC denotes total cholesterol; HDL‐C denotes high‐density lipoprotein cholesterol; MI denotes myocardial infarction.

The selected single‐nucleotide polymorphisms (SNPs), rs693 and rs562338, represent the *ApoB* locus given its pattern of linkage disequilibrium shown in Fig. S1 (Supporting information).

a Percentage is within the genotyped sample.

b Percentage is within each genotype.

c Mean TC and HDL‐C were measured at examination 9 for FHS original cohort because this examination included the largest sample size with nonmissing information on these lipids. For the other FHS cohorts and for the other studies, mean TC and HDL‐C were representatively given at baselines.

Table 1 and Table S1 (Supporting information) show that the proportion of genotypes of rs693 and rs562338 SNPs is virtually the same in each sample. The CHS population was the oldest at baseline, followed by the MESA, ARIC, and FHS. Carriers of each genotype of these SNPs were of about the same age at baseline as well as at death or the end of follow‐up in each sample. The TC and HDL‐C concentrations varied across genotypes and sample. Table 1 and Table S1 (Supporting information) also show the genotype‐specific proportions of myocardial infarction (MI) cases and deaths.

### Associations of genetic variants with lipids

Associations of rs693 and rs562338 representing the *ApoB* locus with endophenotypes (TC and HDL‐C) were evaluated using an additive genetic model with allele A in each SNP as an effect allele. We evaluated the associations of each of these two SNPs, their additive effects, and the associations of each of two polygenic scores (PS‐AA and PS‐GA) with TC and HDL‐C (see Section ‘Experimental procedures’).

#### Associations with TC

Table 2 (Model 1) shows detrimental effects of the rs693_A allele and protective effects of the rs562338_A allele on TC in each study. In most studies, the effects were highly significant. The modest significance for rs693 in MESA was, in part, due to smaller effect size. The detrimental (rs693) and protective (rs562338) effects on TC were consistent in each FHS cohort (Table S2, M1, Supporting information).

**Table 2:** The associations of genetic variants with lipids

Genetic factor, model	ARIC, *N* = 9585			FHS, *N* = 8436			MESA, *N* = 2680			CHS, *N* = 4208			Mega‐analysis, *N* = 24 909		
	β	SE	*P*‐value	β	SE	*P*‐value	β	SE	*P*‐value	β	SE	*P*‐value	β	SE	*P*‐value
Total cholesterol (TC)															
rs693, M1	0.79	0.10	4.2E‐15	0.74	0.11	1.1E‐11	0.44	0.18	1.7E‐02	0.61	0.16	1.0E‐04	0.72	0.06	7.7E‐30
rs562338, M1	−1.16	0.13	2.5E‐18	−1.05	0.14	1.4E‐14	−0.81	0.22	3.0E‐04	−1.18	0.20	3.3E‐09	−1.08	0.08	9.8E‐42
rs693, M2	0.58	0.11	4.0E‐08	0.50	0.12	2.0E‐05	0.25	0.19	2.0E‐01	0.34	0.17	4.2E‐02	0.49	0.07	2.6E‐13
rs562338, M2	−0.92	0.14	2.2E‐11	−0.81	0.15	2.3E‐08	−0.71	0.24	2.7E‐03	−1.04	0.21	9.8E‐07	−0.88	0.09	3.1E‐25
PS‐AA	0.11	0.10	2.3E‐01	0.06	0.11	5.6E‐01	−0.09	0.17	6.2E‐01	−0.11	0.15	4.7E‐01	0.04	0.06	4.8E‐01
PS‐GA	−0.74	0.07	4.0E‐24	−0.69	0.08	2.1E‐18	−0.45	0.13	5.8E‐04	−0.68	0.11	2.1E‐09	−0.69	0.05	2.8E‐51
High‐density lipoprotein cholesterol (HDL‐C)															
rs693, M1	−0.32	0.17	5.6E‐2	−0.41	0.17	1.5E‐2	−0.04	0.29	8.9E‐1	0.16	0.23	5.0E‐1	−0.24	0.10	1.5E‐02
rs562338, M1	0.04	0.22	8.4E‐1	−0.16	0.21	4.5E‐1	−0.19	0.36	5.9E‐1	0.10	0.29	7.5E‐1	−0.04	0.13	7.8E‐01
rs693, M2	−0.34	0.18	5.3E‐2	−0.52	0.18	3.5E‐3	−0.10	0.31	7.4E‐1	0.20	0.21	3.2E‐1	−0.28	0.10	7.4E‐03
rs562338, M2	−0.09	0.23	7.0E‐1	−0.39	0.22	7.6E‐2	−0.23	0.38	5.4E‐1	0.18	0.31	5.6E‐1	−0.15	0.13	2.5E‐01
PS‐AA	−0.27	0.16	9.3E‐2	−0.48	0.17	2.9E‐3	−0.14	0.28	6.0E‐1	0.18	0.22	4.1E‐1	−0.24	0.10	1.0E‐02
PS‐GA	0.19	0.12	1.3E‐1	0.20	0.12	1.0E‐1	−0.03	0.21	8.9E‐1	−0.03	0.17	8.4E‐1	0.13	0.07	6.2E‐02

*N* denotes maximal number of individuals in the analyses with missing information on rs693, rs562338, TC, and HDL‐C excluded.

M1 denotes model 1 with one reference SNP included.

M2 denotes model 2 with both reference SNPs included.

PS‐AA and PS‐GA denote polygenic scores constructed as counts of the rs693_A or rs562338_A and rs693_G or rs562338_A alleles, respectively.

The effect size beta evaluated for 100 × log_10_(TC) and 100 × log_10_(HDL‐C) is the estimate of cumulative genetic effects over multiple examinations using mixed‐effects regression model. Sign of beta indicates direction of the effect in additive genetic models. The effect alleles were as follows: (i) allele(s) A in each SNP and in polygenic score PS‐AA and (ii) alleles G and A in polygenic score PS‐GA.

SE denotes standard error.

These SNPs were also additively associated with TC, although the effects (*P*‐values) were consistently and substantially smaller (larger) than the effects (*P*‐values) of individual SNPs in each study (by 26–44% for rs693 and by 13–30% for rs562338 for effect sizes) and in each FHS cohort (Table 2 and Table S2, M2, Supporting information). Because the number of people in the analyses of individual SNPs and their additive effects were the same, the increase of *P*‐value was due to smaller effect sizes.

Because the effects of the rs693_A and rs562338_A alleles were of opposite signs, the analyses showed highly significant protective effects of the polygenic score PS‐GA, whereas the effects of PS‐AA were small and nonsignificant (Table 2).

Mega‐analysis of all studies showed that the strongest effect per allele could be achieved for rs562338 (β = −1.08, *P* = 9.8 × 10^−42^). This effect was equivalent to decreasing TC concentrations by 5.1 mg dL^−1^ per allele. The most significant effect was, however, observed for the effect alleles in PS‐GA score (β = −0.69, *P* = 2.8 × 10^−51^), which is equivalent to decreasing concentrations of TC by 3.2 mg dL^−1^ per effect allele in a person's genome.

Adjustment of the models by lipid‐lowering treatment resulted in minor improvement of the associations of rs693, rs562338, and PS‐GA with TC (Table S3, Supporting information).

#### Associations with HDL‐C

The rs693_A allele showed detrimental effects on HDL‐C in the ARIC, FHS, and MESA and protective effect in the CHS (Table 2, M1). The effect of this allele was consistently detrimental in each FHS cohort (Table S2, M1, Supporting information). Unlike TC, however, these effects were weak and attain only suggestive effect (ARIC) and conventional (FHS) significances. Neither consistent nor suggestive effects on HDL‐C were seen for the rs562338_A allele (Table 2 and Table S2, M1, Supporting information).

The analyses of additive associations of rs693 and rs562338 with HDL‐C strengthened the effects of the rs693_A and rs562338_A alleles in each study (Table 2, M2) and in each FHS cohort (Table S2, M2, Supporting information). The effect direction for the rs562338_A allele became consistently negative in the ARIC, FHS, and MESA (Table 2 and Table S2, Supporting information), but not in the CHS (Table 2). The detrimental effect of the rs562338_A allele attained suggestive effect significance in FHS.

Polygenic scores PS‐AA and PS‐GA showed at most minor strengthening of the effects in individual studies.

Due to mixed effect directions, mega‐analysis of all studies did not improve the best estimate in a specific study for each SNP and PS‐AA. The improved *P*‐value for PS‐GA (β = 0.13, *P* = 6.2 × 10^−2^) was due to the dominant role of the rs693_G allele.

Adjustment of the models by lipid‐lowering treatment did not affect the associations of rs693, rs562338, and PS‐AA with HDL‐C (Table S4, Supporting information).

The analyses of the potential mediating role of TC in the associations of rs693 and rs562338 with HDL‐C showed that TC modulated the effects of rs693 on HDL‐C that resulted in decreasing *P*‐values and strengthening the effect sizes in the FHS and ARIC (Table S5, Supporting information). This modulating effect implies that rs693 is associated with TC and HDL‐C additively in these studies. The potential protective association of rs562338 with HDL‐C in FHS was, however, partly mediated by TC. No other significant associations were observed.

### Lipid‐unadjusted associations of rs693 and rs562338 with risks of MI

Table 3 (M1) shows the protective associations of the rs693_A allele with risks of MI in ARIC (β = −0.08, *P* = 0.13) and in the pooled sample of the FHS original and FHSO cohorts (β = −0.18, *P* = 1.8 × 10^−3^). Table S6 (M1, Supporting information) also shows protective effects in each of these FHS cohorts. In the FHS 3rd Gen cohort, the effect was detrimental (β = 0.36, *P* = 0.27) (Table S6, M1, Supporting information). However, this cohort included only 19 MI cases, and therefore, the analyses using this small sample were unreliable.

**Table 3:** The associations of rs693 and rs562338 with risks of myocardial infarction

Genetic factor, model	ARIC *N*_T/C_=9562/764			FHS^a^ *N*_T/C_ = 4485/587			MESA *N*_T/C_ = 2505/75			CHS *N*_T/C_ = 4196/931		
	β	SE	*P*‐value	β	SE	*P*‐value	β	SE	*P*‐value	β	SE	*P*‐value
Not adjusted for lipids												
rs693, M1	−0.08	0.05	1.3E‐1	−0.18	0.06	1.8E‐3	0.19	0.17	2.5E‐1	0.13	0.05	6.9E‐3
rs562338, M1	−0.09	0.07	2.2E‐1	0.04	0.07	5.8E‐1	−0.01	0.21	9.5E‐1	−0.02	0.06	8.0E‐1
rs693, M2	−0.11	0.06	4.8E‐2	−0.19	0.06	1.7E‐3	0.21	0.18	2.3E‐1	0.14	0.05	5.6E‐3
rs562338, M2	−0.13	0.07	7.5E‐2	−0.04	0.08	5.8E‐1	0.07	0.22	7.4E‐1	0.04	0.06	5.0E‐1
Adjusted for total cholesterol												
rs693, M1	−0.10	0.05	5.2E‐2	−0.23	0.06	6.1E‐5	0.18	0.17	2.8E‐1	0.13	0.05	7.3E‐3
rs562338, M1	−0.05	0.07	4.9E‐1	0.10	0.07	1.8E‐1	−1E‐6	0.21	1.0E‐0	−0.01	0.06	8.2E‐1
rs693, M2	−0.12	0.06	2.4E‐2	−0.23	0.06	1.5E‐4	0.20	0.17	2.6E‐1	0.14	0.05	5.9E‐3
rs562338, M2	−0.10	0.07	1.8E‐1	−0.005	0.08	9.5E‐1	0.08	0.22	7.1E‐1	0.04	0.06	4.9E‐1
Adjusted for total and high‐density lipoprotein cholesterols												
rs693, M1	−0.12	0.05	2.4E‐2	−0.24	0.06	3.6E‐5	0.16	0.17	3.4E‐1	0.13	0.05	7.7E‐3
rs562338, M1	−0.05	0.07	4.5E‐1	0.10	0.07	1.8E‐1	0.02	0.21	9.3E‐1	0.001	0.06	9.8E‐1
rs693, M2	−0.15	0.06	8.9E‐3	−0.24	0.06	8.7E‐5	0.19	0.18	3.0E‐1	0.14	0.05	4.6E‐3
rs562338, M2	−0.11	0.07	1.3E‐1	−0.007	0.08	9.2E‐1	0.09	0.22	6.7E‐1	0.06	0.07	3.3E‐1

*N*_T/C_ denotes total number (T) of individuals in the analyses and the number of cases (C) among them.

M1 denotes model 1 with one reference SNP included.

M2 denotes model 2 with both reference SNPs included.

The effect beta was evaluated in the Cox proportional hazard regression model. Sign of beta indicates direction of the effect in additive genetic models with alleles A considered as effect alleles for each SNP.

SE denotes standard error.

a The 3rd generation cohort of the Framingham Heart Study (FHS) was not included because of small number of MI events (*N* = 19).

The same rs693_A allele also showed detrimental effects in the other two studies, MESA (β = 0.19, *P* = 0.25) and CHS (β = 0.13, *P* = 6.9 × 10^−3^). Unlike the FHS 3rd Gen cohort, the MESA and CHS included substantially larger number of MI cases (Table 3).

Neither the associations of rs562338 with risks of MI were significant in each sample (Table 3 and Table S6, M1, Supporting information) nor were the effect directions consistent across different studies (Table 3, M1).

Additive effects of rs693 and rs562338 attained conventional (rs693) and suggestive effect (rs562338) significance in ARIC (Table 3, M2). The analyses also showed that the effects of these two SNPs in the other samples were virtually independent.

The associations of rs693 and rs562338 with risks of MI were not altered when only prospective MI cases were considered (Table S7, Supporting information).

**Figure 1:**
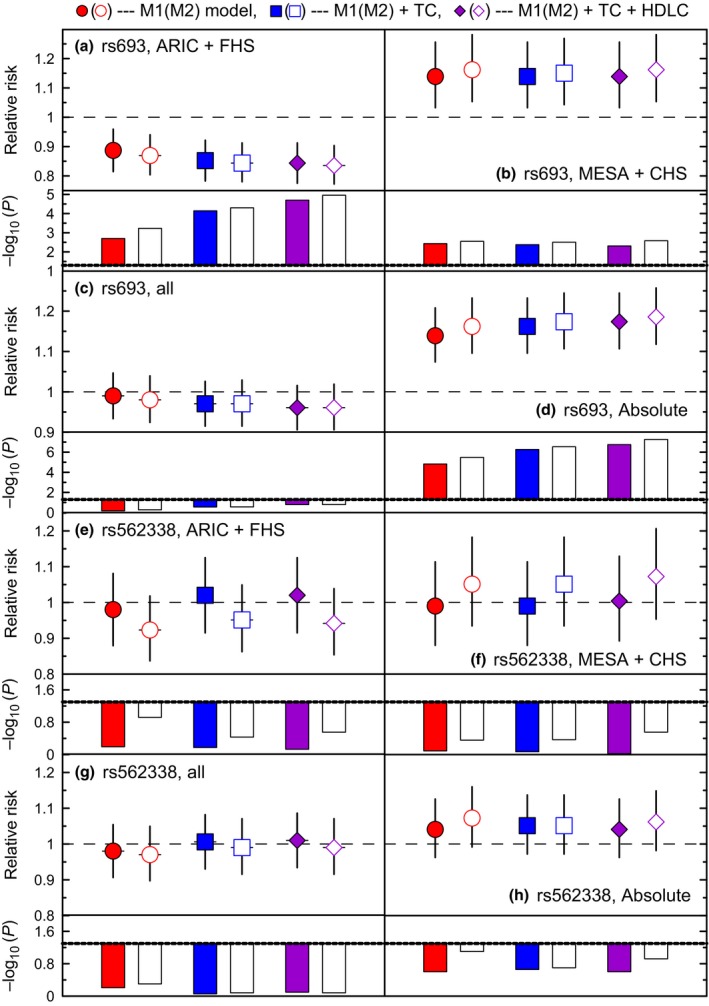
Pooled associations of rs693 and rs562338 with the risks of MI. Symbols show relative risks (RRs) of MI for each SNP separately (M1, filled symbols) and SNPs additive effects (M2, open symbols). The analyses were not conditioned on lipids (dots) and conditioned on TC (squares), and TC and HDL‐C (diamonds). Vertical lines show 95% confidence intervals. Bars show −log_10_(*P*‐value). Dotted line shows cutoff for significance −log_10_(0.05) = 1.3. Figures show the results pooling (a, e) ARIC and FHS, (b, f) MESA and CHS, (c, g) all studies following GWAS, and (d, h) all studies disregarding the effect directions. Other details are in Table S8 (Supporting information).

Mega‐analysis of all studies, which resembled the conventional strategy of pooling the effects in GWAS meta‐analyses, showed weak nonsignificant effects of each SNP on risks of MI (Fig. 1c,g). Mega‐analysis of the studies supporting the protective and detrimental effects of rs693 separately revealed highly significant protective (β = −0.14, *P* = 5.9 × 10^−4^) and detrimental (β = 0.15, *P* = 2.8 × 10^−3^) effects (Table 8, Supporting information, M2, no lipid adjustment). These effects imply relative risk RR=0.87 (Fig. 1a, M2) and RR = 1.16 (Fig. 1b, M2). Evaluating pooled effects in all studies disregarding the effect directions, the overall significance of the effect for rs693 (or, equivalently, the probability of false finding) was *P* = 3.3 × 10^−6^.

### The role of lipids and lipid‐lowering treatment in the associations of rs693 and rs562338 with MI

Here, we examined whether endophenotypes (lipids) could mediate the associations of rs693 and rs562338 with risks of MI.

We observed two qualitatively different patterns depending on the direction of the effects of rs693 in each study. First, adjustment of the models by TC, and by TC and HDL‐C consistently strengthened the protective effect of rs693 against MI in ARIC and FHS (Table 3). For example, the protective effect of rs693 in ARIC increased by about 50% and attained conventional significance, that is, β = −0.08, *P* = 0.13 vs. β = −0.12, *P* = 2.4 × 10^−2^ (Table 3).

Second, adjustment of the models by TC, and by TC and HDL‐C, virtually did not change the estimates for rs693 in MESA and CHS (Table 3).

Adjustment of the models by lipids showed at most a minor mediating effect of lipids on the associations of rs562338 with risks of MI in ARIC and FHS and no influence in MESA and CHS. The effects of this SNP remained nonsignificant in each study.

The modulating effect of lipids substantially improved the estimates for rs693 in mega‐analyses (Fig. 1a). For example, the protective effect of the rs693_A allele in the pooled FHS and ARIC sample was improved from β = −0.14, *P* = 5.9 × 10^−4^ to β = −0.18, *P* = 1.1 × 10^−5^ (Table S8, Supporting information), that is equivalent to improving relative risks from RR = 0.869 to RR = 0.835 (Fig. 1a). Disregarding the effect direction, the effect for rs693 in all datasets virtually attained the level of genomewide significance, *P* = 5.5 × 10^−8^.

The analyses of the role of lipid‐lowering treatment showed that this treatment did not change the associations of rs693 and rs562338 with risks of MI (Table S9, Supporting information).

### Associations of rs693 and rs562338 with risks of overall mortality

Here, we evaluated the individual and additive effects of rs693 and rs562338 on risks of overall death and the potential mediating role of lipids and MI (Table S10, Supporting information).

Neither individual nor additive significant effects were observed in ARIC and FHS for these SNPs in models not adjusted for lipids and MI. In MESA, the rs693_A allele decreased risks of death (β = −0.24, *P* = 3.1 × 10^−2^) whereas the rs562338_A allele increased risks of death (β = 0.26, *P* = 4.2 × 10^−2^). However, the effects of these alleles were partly overlapping, making their additive estimates nonsignificant. In CHS, the rs693_A allele increased risks of death individually (β = 0.06, *P* = 6.7 × 10^−2^) and additively (β = 0.07, *P* = 4.0 × 10^−2^). No significant effect for rs562338 was seen in CHS.

Adjustments of the models by lipids improved the protective effect of the rs693_A allele in ARIC, allowing it to attain marginal significance (β = −0.08, *P* = 5.6 × 10^−2^). This effect was linearly independent of rs562338. Although this adjustment also somewhat improved the protective effect of rs693 in the FHS, this effect did not attain significance. In MESA and CHS, lipids virtually did not affect the estimates.

Conditioning the analyses by lipids and MI showed at most a minor mediating role of MI in the effects of rs693 and rs562338 on risks of overall death.

### Lipid‐adjusted additive associations of rs693 and rs562338 with risks of mortality for MI patients

The rs693_A and rs562338_A alleles showed protective effects on risks of mortality for MI patients who died during the follow‐up in ARIC and FHS regardless of whether they were contrasted by MI survivors and individuals without MI or by individuals without MI only (Table 4). The effect was highly significant in FHS for rs693 (β = −0.30, *P* = 9.6 × 10^−5^). In MESA and CHS, we observed detrimental effects of these alleles, although MESA had small number of events.

**Table 4:** The associations of rs693 and rs562338 with risks of mortality for MI patients

Genetic factor, model	ARIC, *N*_C_ = 236			FHS^a^, *N*_C_ = 375			MESA, *N*_C_ = 19			CHS, *N*_C_ = 637		
	β	SE	*P*‐value	β^a^	SE	*P*‐value	β^a^	SE	*P*‐value	β^a^	SE	*P*‐value
rs693, M21	−0.14	0.10	1.6E‐1	−0.29	0.08	1.7E‐4	0.43	0.36	2.3E‐1	0.12	0.06	4.5E‐2
rs562338, M21	−0.21	0.13	1.1E‐1	−0.13	0.10	2.1E‐1	0.71	0.40	7.1E‐2	0.06	0.08	4.2E‐1
rs693, M22	−0.14	0.10	1.6E‐1	−0.30	0.08	9.6E‐5	0.43	0.36	2.3E‐1	0.13	0.06	3.0E‐2
rs562338, M22	−0.22	0.13	1.0E‐1	−0.13	0.10	1.8E‐1	0.70	0.39	7.3E‐2	0.07	0.08	4.0E‐1

M21 denotes the model with cases defined as death among MI patients, and the others were considered as controls. The model included both reference SNPs and was adjusted for basic covariates, TC, and HDL‐C. The total samples were as follows: ARIC, *N*_T_ = 9562; FHS, *N*_T_ = 4485; MESA, *N*_T_ = 2505; CHS, *N*_T_ = 4196.

M22 was the same as M21 except the controls were individuals with no MI. The total samples were as follows: ARIC, *N*_T_ = 9034; FHS, *N*_T_ = 4273; MESA, *N*_T_ = 24494; CHS, *N*_T_ = 3902.

The number of cases (*N*_C_) in the analyses is given in the table.

a The 3rd generation cohort of the Framingham Heart Study (FHS) was not included because of small number of deaths after MI events (*N* = 5).

The directions of these effects were consistent with the directions of the effects for these two SNPs on MI in each dataset (Table 3, M2).

**Figure 2:**
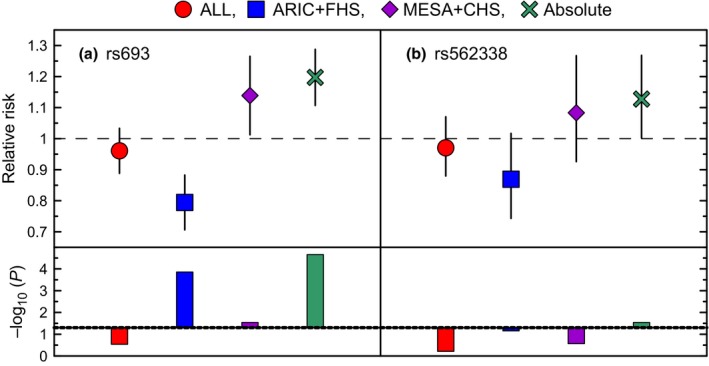
Pooled associations of rs693 and rs562338 with the risks of mortality for MI patients. Symbols show the additive relative risks (RRs) of mortality for MI patients compared to the others. The results are shown pooling all studies following GWAS (dots), ARIC, and FHS (squares), MESA and CHS (diamonds), and all studies disregarding the effect directions (crosses). Vertical lines show 95% confidence intervals. Bars show −log_10_(*P*‐value). Dotted line shows cutoff for significance −log_10_(0.05) = 1.3. Other details are in Table S11 (Supporting information).

Mega‐analysis of the ARIC and FHS revealed a highly significant protective effect of rs693 (β = −0.24, *P* = 8.3 × 10^−5^) and a marginally significant effect of rs562338 (β = −0.15, *P* = 5.9 × 10^−2^) (Table S11, Supporting information). This implies that each copy of the rs693_A allele reduces risks of mortality for MI patients compared to individuals without MI by about 21% (RR = 0.79, Fig. 2a). The detrimental effects of rs693 and rs562338 in MESA and CHS are substantially smaller than the protective ones (Fig. 2). Only the effect for rs693 attained conventional significance (β = 0.14, *P* = 1.9 × 10^−2^) (Table S11, Supporting information).

Due to opposite effect directions, pooled estimates resembling conventional GWAS meta‐analyses were nonsignificant, *P* = 0.31 for rs693 and *P* = 0.57 for rs562338. Evaluating pooled effects in all studies disregarding the effect directions, the probability of false finding was *P* = 3.5 × 10^−5^ for rs693 and *P* = 1.5 × 10^−2^ for rs562338 (Fig. 2).

## Discussion

In this article, we examined the associations of rs693 and rs562338 SNPs representing the *ApoB* locus with endophenotypes (TC and HDL‐C) and phenotypes (MI and survival) in four studies, ARIC, FHS, MESA, and CHS. The analyses followed an analytic strategy wherein we evaluated the following:

1. the associations of rs693 and rs562338 and polygenic scores constructed using these SNPs with TC and HDL‐C, and potential mediating/modulating role of lipid‐lowering therapy and TC.
2. the associations of rs693 and rs562338 with risks of MI, and potential mediating/modulating role of lipids and lipid‐lowering therapy.
3. the associations of rs693 and rs562338 with survival, and potential mediating/modulating role of lipids, lipid‐lowering therapy, and MI.

This analytic strategy is driven by a more realistic concept of genetic predisposition to inherently heterogeneous, age‐related traits (explicated in the Introduction).

Table 5 illustrates the types of the observed associations discussed in Subsections (ApoB and lipids)‐(ApoB and MI) below.

**Table 5:** Summary of the effects of rs693 and rs562338 on endophenotypes (TC and HDL‐C) and a phenotype (MI)

Study	rs693_A				rs562338_A			
	TC	HDL‐C	MI	Lipids and MI	TC	HDL‐C	MI	Lipids and MI
FHS	D	D	P	improves protective effect	P	D^a^	P^a^	at most minor mediator
ARIC	D	D^a^	P	improves protective effect	P	D^a^	P^a^	at most minor mediator
MESA	D	D^a^	D^a^	neutral	P	D^a^	D^a^	neutral
CHS	D	P^a^	D	neutral	P	P^a^	D^a^	neutral

Symbol ‘D’ (‘P’) denotes detrimental (protective) effect.

For TC ‘D’ (‘P’) means that allele ‘A’ is associated with increased (decreased) TC concentrations.

For HDL‐C ‘D’ (‘P’) means that allele ‘A’ is associated with decreased (increased) HDL‐C concentrations.

For risks of MI, ‘D’ (‘P’) means that allele ‘A’ is associated with increased (decreased) MI risks.

Columns ‘lipids and MI’ show the role of lipids in the associations of a given allele with MI.

a The effect is nonsignificant.

### ApoB and lipids

Consistently with prior GWAS meta‐analyses (Willer *et al*., 2008; Aulchenko *et al*., 2009; Sabatti *et al*., 2009), our analyses show strong detrimental (rs693_A allele) and protective (rs562338_A allele) associations with TC in each sample, that is, in each study (Table 2) and in each Framingham cohort (Table S2, Supporting information). Further, our analyses document additive effects of the rs693_A and rs562338_A alleles although they are consistently and substantially smaller than individual effects of these SNPs in each sample.

Mega‐analyses show that the best estimate of the effect size per effect allele is achieved for rs562338, whereas the best significance is for polygenic score PS‐GA. The absolute effect size for rs562338 is about 57% larger than that for PS‐GA and 50% larger than that of rs693 (Table 2) suggesting a more prominent role of rs562338 (or other causal variant(s) which this SNP is a proxy for) in lipid metabolism than rs693.

Unlike the effects of rs693 and rs562338 on TC, the effects of these SNPs on HDL‐C are inconsistent across studies and at most minor, although a somewhat stronger effect on HDL‐C is observed for rs693. This SNP may additively influence TC and HDL‐C.

Lipid‐lowering therapy showed at most tiny modulating effect on the associations of rs693 and rs562338 with lipids.

### ApoB and MI

Unlike the strong and consistent detrimental (rs693_A allele) and protective (rs562338_A allele) associations with TC in all four studies, the associations of the same alleles with MI are substantially more complex and are not necessarily in line with the associations with TC (Table 5), despite the compelling role of lipids in cardiovascular health (Law *et al*., 2003; Clarke *et al*., 2007).

Contrary to the detrimental effect of the rs693_A allele on TC, the same allele shows protective effects against MI in two independent studies, the ARIC and FHS, which include a large number of MI events. This MI‐protective effect is strong and significant in the pooled ARIC and FHS sample (β = −0.14, *P* = 5.9 × 10^−4^) implying that the risk of MI is decreased by about 13% per the rs693_A allele (Fig. 1a, open dot). Controlling for lipids substantially and consistently improves the association of the rs693_A allele with risks of MI in each study (Table 4) and in the pooled ARIC and FHS sample (β = −0.18, *P* = 1.1 × 10^−5^). This leads to decreasing the risk of MI by about 30%, improving the protective effect from 14% to about 17% per the rs693_A allele (Fig. 1a, open diamond). This implies that the effect allele homozygotes are at about 34% smaller risk of MI than the noncarriers of the effect allele.

Consistently with the detrimental effect of the rs693_A allele on TC, this allele also shows detrimental effects on MI in two other independent studies, the MESA and CHS, which also include a large number of MI events. The MI‐detrimental effect in the pooled MESA and CHS sample (β = 0.15, *P* = 2.8 × 10^−3^) is as strong as the MI‐protective effect in the pooled ARIC and MESA sample. It implies about a 14% increase of the risk of MI per the rs693_A allele (Fig. 1b, open dot). Contrary to common expectation, this MI‐detrimental effect is not mediated by lipids (Fig. 1b).

Despite the strong protective effect of the rs562338_A allele on TC, its effect on MI is small and nonsignificant in the ARIC and FHS combined, β = −0.02, *P* = 0.64 (Table S8, Supporting information). The protective effect of this allele improves by controlling for the effect of rs693 (β = −0.08, *P* = 0.12); however, it does not attain significance (Fig. 1e, open dot). The analyses show at most minor role of lipids as mediators of this protective nonsignificant effect against MI (Fig. 1e).

Contrary to the protective effect of rs562338_A allele on TC, we observed detrimental effects of this allele on risks of MI in the pooled MESA and CHS sample, which were nonsignificant. Lipids virtually did not mediate this effect either (Fig. 1f).

The lipid‐lowering therapy did not play a noticeable role in the observed associations.

Thus, our results provide three important insights. First, despite the more prominent role of rs562338 in regulation of TC, rs693 has a substantially more prominent role in MI than rs562338. Second, rs693 and lipids influence the risk of MI additively and lipids virtually do not mediate the effect of rs693 on MI (Table 5). Third and most importantly, the rs693_A allele influences the risk of MI antagonistically in different populations.

The antagonistic effects are a robust finding because they are observed in samples with large and about the same number of MI events and each effect is replicated in independent studies. They imply that different mechanisms mediate the association of rs693 with the risk of MI. One mechanism is likely related to lipid metabolism because the additive effect of lipids improves (but not mediate) the MI‐protective effects (called lipid‐sensitive mechanism) in ARIC and FHS. The other mechanism is not sensitive to lipids because the additive effect of lipids does not affect the MI‐detrimental effects (called lipid nonsensitive mechanism) in MESA and CHS. Validation of these two mechanisms in independent studies is another robust support to the observed antagonistic effects. Indeed, antagonistic effects are not exceptions and their importance is recognized elsewhere (Schnebel & Grossfield, 1988; Williams & Day, 2003; van Heemst *et al*., 2005; Alexander *et al*., 2007; Kulminski *et al*., 2013).

### ApoB and survival

The effects of rs693 and rs562338 on overall mortality were small and attained only marginal significance in some studies. The effects of these SNPs on risks of mortality for MI patients, however, were more pronounced (Fig. 2). Furthermore, the protective effects of the rs693_A and rs562338_A alleles on MI in ARIC and FHS were translated into reduction of the risk of mortality for MI patients. Likewise, the detrimental effects of the rs693_A and rs562338_A alleles on MI in MESA and CHS were translated into increase of the risk of mortality for MI patients. The lack of significant effects of these alleles on overall mortality and the presence of significant effects on mortality for MI patients implies that these SNPs can contribute to MI‐specific mortality and non‐MI mortality in an antagonistic manner.

### Implications for studies of genetic predisposition to healthspan and lifespan

Our results show that the conventional GWAS strategy for the analyses of traits relevant to healthspan and lifespan is problematic and can be misleading. This strategy can also heavily underuse available resources.

Indeed, GWAS implicitly assumes that genetic predisposition to endophenotypes can be translated into predisposition to downstream phenotypes (Willer *et al*., 2008). Following our analytic approach, we show that this may not be the case (see Subsections ApoB and MI and ApoB and survival above) even in the most favorable situation when genetic variants have been likely fixed by evolutionary selection related to endophenotypes. According to our results, *rigorous* tests of the hypotheses on connections of genes with endophenotypes and phenotypes are imperative. Without rigorous tests, the analyses may overlook complex mechanisms of connections of genetic variants with endophenotypes and phenotypes that may lead to misleading conclusions. The latter is demonstrated in the present study by antagonistic effects of the same allele in different populations.

A ‘side effect’ of overlooking complex modes of gene actions is the underuse of available resources. Indeed, following the conventional GWAS strategy and pooling the effects from all four studies, the best estimate of the risks of MI in our data was for rs693, RR = 0.96, *P* = 0.16 (Fig. 1c, open diamond) in the sample of 20 748 individuals with 2357 MI events. Given this small effect, it is traditionally argued that even larger samples are needed to improve power. Explicating antagonistic effects of this SNP, we see that this small effect is actually a superposition of strong effects of opposite directions with overall significance reaching virtually genomewide level, *P* = 5.5 × 10^−8^ (Fig. 1d, open diamond). This significance highlights very small probability of false finding. The difference in *P*‐values (*P* = 5.5 × 10^−8^ vs. *P* = 0.16) shows that our analytic strategy substantially outperforms the conventional GWAS strategy.

The significant estimate (*P* = 5.5 × 10^−8^) is contrasted by, and the nonsignificant estimate (*P* = 0.16) is aligned with, the small effects of rs693 on MI and coronary artery disease (CAD) and/or tiny significance in other studies. For example, conventional GWAS meta‐analysis (Willer *et al*., 2008) reported weak detrimental effect of the rs693_A allele on CAD/MI (odds ratio [OR] = 1.07, *P* = 0.028) in the sample with slightly smaller number of CAD/MI events than in the present study. Importantly, meta‐analysis of the results of mostly candidate‐gene studies supported the protective effect of the rs693_A allele on CAD/MI (OR=0.83, *P* = 0.06) (Xiao *et al*., 2015).

### The role of rs693 and lipids in MI pathogenesis

Our results provide two insights on the role of rs693 and lipids in MI pathogenesis.

The first is based on the observation of discordant effects of the rs693_A allele on lipids and the risks of MI (and mortality for MI patients) and on the observation of the lack of a mediating role of lipids in the effects of this allele on MI (see Table 5 and Subsections ApoB and MI and ApoB and survival above). These observations suggest that TC (and LDL‐C because TC is proxy for LDL‐C) may not be pathogenic for carriers of this allele.

The second is based on the observation of improvement of the protective effect of rs693_A on MI by conditioning on lipids (Fig. 1a). This result implies that carriers of the rs693_A allele have smaller risks of MI at increased concentrations of TC and HDL‐C. This is not surprising for HDL‐C because of the beneficial role of HDL‐C in cardiovascular (CV) health. However, this is surprising for TC because increased TC concentrations are established risk factors for CV events. These observations suggest that increased TC concentrations can enhance protection of the rs693_A carriers against MI.

A plausible mechanism could be that modified LDL‐C, rather than native LDL‐C, is an important predictor of CAD/MI. For example, oxidation of LDL‐C (oxLDL) and the resulting modification of the ApoB‐100 lysine groups within the arterial walls play a role in atherogenesis and atherothrombosis (Mertens & Holvoet, 2001). If this is the case, the TC‐increasing effect of the rs693_A allele may not result in increased risks of MI because LDL‐C may not correlate with oxLDL (Ehara *et al*., 2001). The lack of such correlation was suggested to explain findings in blacks who have better lipid profiles but higher rates of CV‐related conditions (Tsimikas *et al*., 2009). The role of oxidation is also supported by antioxidant properties of HDL‐C and by capacity of unmodified HDL‐C to protect LDL‐C from oxidation (Mertens & Holvoet, 2001). Advanced glycation end product‐modified LDL (AGE‐LDL) is another modification that can promote atherosclerosis, particularly in diabetes. AGE‐LDL is sensitive to oxidation and may be an important risk factor for the development of MI (Hodgkinson *et al*., 2008). Both oxLDL and AGE‐LDL are capable of inducing proinflammatory events contributing to atherogenesis through the activation of pathways associated with innate immunity (Hodgkinson *et al*., 2008; Tall & Yvan‐Charvet, 2015).

Given the beneficial role of the rs693_A allele for evolutionary selection (see next Subsection), the oxidative mechanism may be evolutionarily adapted to specific environments. Then, the observed antagonistic effects of the rs693_A allele should reflect differences in evolutionary‐related exposures in different populations. If modified LDL‐C plays a central role in pathogenesis of CAD/MI, it is possible that antioxidants could modulate oxidatively modified LDL‐C and, thus, explain the observed antagonistic effects. It is clear, however, that detailed studies of potential mechanisms of beneficial effects of increased TC concentrations for carriers of the rs693_A allele are needed, particularly, because this increase may work as a compensatory mechanism to balance decline in immune function with age (Ravnskov, 2003; Tall & Yvan‐Charvet, 2015).

### Genetic health risks and evolution

The famous maxim by Theodosius Dobzhansky, ‘Nothing in biology makes sense except in the light of evolution’, highlights the importance of evolutionary constraints in genetic effects on health outcomes (Dobzhansky, 1973), especially those, which are characteristic for the postreproductive period. The effect alleles ‘A’ in rs693 and rs562338 are considered as derived alleles, which have been fixed during evolutionary adaptation. For example, rs693 is believed to be fixed by the evolutionary adaptation to ecoregions and climate (Hancock *et al*., 2010). The strong role of evolutionary selection in fixing rs693 is supported by the nearly 50% frequency of the derived allele. A role of evolution in these polymorphisms is also supported by consistent patterns of linkage disequilibrium (LD) in the *ApoB* locus in different populations of whites, by consistent LD patterns in blacks, and by substantial differences of the LD patterns for whites and blacks (Fig. S1, Supporting information). Strong and consistent associations of rs693 and rs562338 with TC in all study populations of whites considered in the present work may therefore indicate involvement of lipid metabolism in the evolutionary adaptation affecting these SNPs. However, despite the likely role of evolution in fixing the rs693_A and rs562338_A alleles, our results on discordant effects of these alleles on TC and MI (Table 5) do not support the hypothesis that these SNPs are involved in pathological dysregulation of lipids causing MI. This necessarily implies that even if the risk alleles have been selected against some endophenotypes, these alleles may affect downstream phenotypes through different mechanisms or may not affect them at all.

## Conclusions

Our analyses confirm strong and robust associations of rs693 and rs562338 SNPs representing the *ApoB* locus with endophenotype (TC). However, the analyses highlight a nontrivial role of these SNPs (or other causal variant(s) which these SNPs are proxy for) in downstream phenotypes (MI and survival). Most importantly, we show that (i) genetic predisposition to TC is not translated into predisposition to MI and survival, (ii) the rs693_A allele influences risks of MI and mortality for MI patients additively with lipids, (iii) the rs693_A allele influences these risks in an antagonistic manner in different populations, and (iv) increased TC concentrations can be beneficial for carriers of the rs693_A allele. These results uncouple the influence of the same alleles on endophenotype and phenotypes despite a potential causal relationship among the latter. This holds for the risk alleles, which are likely fixed by evolutionary selection related to endophenotypes. Our analytic strategy, which rigorously addresses logistics of connections between the *ApoB*‐related variants, endophenotypes (lipids) and phenotypes (risks of MI and survival) in evolutionary context, substantially outperforms the traditional GWAS strategy by explicating the complex modes of action of genetic variants. This reveals the highly significant association of rs693 with MI (*P* = 5.5 × 10^‐8^) that is contrasted by the weak estimate following the GWAS strategy (*P* = 0.16) in the same sample. Our results imply that the traditional GWAS strategy should be used with caution and should be considered as a preliminary step to gain insights on genetic predisposition to healthspan and lifespan.

## Experimental procedures

### Data

*The ARIC study* (Sharrett, 1992) participants (aged 45–64 at baseline in 1987) were randomly selected at four field centers across the United States. Measurements of TC and HDL‐C were available at 4 visits. Data on onsets of MI and survival were available through 2004. Genotyping in 12 771 ARIC participants (*N* = 9633 whites) was conducted using Affymetrix 6.0 array (1000 K SNPs).

*The FHS* (Cupples *et al*., 2009). We used data from 28 visits of the FHS original cohort (aged 28–62 years at baseline in 1948), 8 visits of the FHS Offspring (FHSO) cohort (aged 5–65 years at baseline in 1970), and one visit of the 3rd generation (3rd gen) cohort (aged 21–71 at baseline in 2001). Measurements of TC and HDL‐C were available at multiple visits in the FHS/FHSO and the baseline in the 3rd Gen cohort. Data on onsets of MI and survival were available through 2011. Genotyping of 9167 FHS participants was done using Affymetrix 500K array (Cupples *et al*., 2009).

*The MESA* (Bild *et al*., 2002) participants (aged 43–83 at baseline in 2000) were recruited from six field centers across the United States. Measurements of TC and HDL‐C were available at 5 visits. Data on onsets of MI and survival were available through 2012. Genotyping of 8224 MESA participants (*N* = 2686 whites) was conducted using Affymetrix 6.0 array.

*The CHS* (Fried *et al*., 1991). The CHS participants (*N* = 5201, mostly whites) aged 65+ years at baseline in 1989 were examined annually through 1999. After June 1999, two phone calls per year to participants collected information on incidence of diseases and death. Deaths also were ascertained through surveillance and at semi‐annual contacts. Data on onsets of MI and survival were available through 2004. Genotyping of 4435 whites was done using Illumina CVDSNP55v1_A array.

### Endophenotypes and phenotypes

Lipids were considered as endophenotypes. They included TC as a proxy for LDL‐C (LDL‐C was not available for large number of individuals in the FHS) and HDL‐C. Phenotypes included MI and death.

### Genetic variants

The analyses were focused on rs693 and rs562338 SNPs representing the *ApoB* locus in the available data (Fig. S1a, Supporting information). The rs693 is a synonymous variant in the *ApoB* gene and rs562338 is an intergenic SNP, which is about 21‐kb downstream of *ApoB*. Individuals with a genotypic missingness of > 5% were excluded in each study. For the FHS study, individuals in families and families with Mendel error rates above 2% were excluded. The selected SNPs were in weak linkage disequilibrium (LD) with *r* ^2^ equal to about 0.1 in each study.

Alleles rs693_A and rs562338_A (Table 1) were considered effect alleles in all statistical tests for these SNPs. We also constructed two polygenic scores by counting the number of specific alleles from these SNPs in a given individual. One polygenic score (PS‐AA) was constructed by counting the alleles A in these SNPs; these alleles were considered effect alleles. The other score (PS‐GA) was constructed by counting the rs693_G and rs562338_A alleles; these alleles were considered effect alleles.

### Analysis

We evaluated the associations for each of these two SNPs, their additive effects, and, when warranted, the effects of each of the two polygenic scores in whites. The analyses were conducted using an additive genetic model.

Associations with lipids were characterized by a mixed‐effects linear regression model (*lme4* package in R). Measurements of lipids were log‐transformed (and multiplied by 100 for better resolution) to offset potential bias due to skewness of their frequency distributions. The TC and HDL‐C in each study were measured longitudinally. We evaluated the associations for SNPs given measurements of lipids for individuals of a given age at each examination with available measurements. We used a three‐level mixed‐effects regression model to account for familial and repeated‐measurements correlations. Information on longitudinal measurements has multiple advantages including statistical power gain in the analyses (Shi *et al*., 2009).

The risks of MI and death were evaluated using the Cox proportional hazard mixed‐effects regression model (*coxme* package in R) to adjust for potential clustering. Information on both prospective and retrospective onsets of MI in the FHS and CHS was used in these analyses. The use of retrospective onsets in a failure‐type model is justified by Prentice & Breslow (1978). These analyses provide estimates of the effects in a given population. We tested whether or not the estimates were altered by considering prospective onsets only. The time variable in the Cox regression analyses was the age at onset of a trait or the age at right censoring.

Risks of MI in the models conditional on lipids were evaluated using lipids measured at baselines (in the FHS original cohort, the model was fitted starting at visit 9 at which sufficiently large number of individuals with measured lipids was available). A limitation of this approach, which is commonly adopted to offset the problem of reverse causation, is that it consider cross‐sectional measurements of lipids, whereas their concentrations, and possibly effects on CV events, can change over the life course (Yashin *et al*., 2006; Felix‐Redondo *et al*., 2013). Thus, dynamic aspect of the problem can be important. Nevertheless, the adopted static approach makes sense because changes in lipid concentrations may not necessarily affect genetic associations with these traits (Kulminski *et al*., 2013) due to their polygenic and heterogeneous nature.

All statistical tests were adjusted for the following: (all studies) age, sex, (ARIC and MESA) field center, (FHS) FHS cohorts, and whether the DNA samples had been subject to whole‐genome amplification (Ikram *et al*., 2009). We did not adjust statistical tests for lipids by fasting because (i) information on fasting was not available for most examinations in the FHS original cohort and (ii) according to increasing evidence, including from large‐scale studies, fasting may not substantially change levels of lipids (Sidhu & Naugler, 2012). We did not adjust statistical tests for lipids by lipid‐lowering treatment. Instead, we examined whether this treatment (defined as taken lipid‐lowering drugs or not) modulated the associations with lipids and risks of MI and death.

Pooled estimates were evaluated by mega‐analysis when individuals, rather than the results, from different studies were pooled in one sample, when possible. These analyses were adjusted for intrastudy covariates and interstudy differences. Meta‐analysis of the estimated effects was conducted using the fixed‐effects model implemented in the *metafor* package in R. Meta‐analysis was conducted when the proportionality assumption in the Cox model was violated.

## Author contributions

A.M.K. conceived and designed the experiment and wrote the manuscript; Y.K., Y.L., O.B., M.D., L.A., and D.W. prepared data and conducted statistical analyses; I.C. and K.G.A. conducted the experiment; S.V.U., E.S., and A.I.Y. discussed the results and drafted the manuscript

## Funding

This research was supported by Grants No P01 AG043352 and R01AG047310 from the National Institute on Aging.

## Conflict of interest

The authors declare no conflict of interest.

## Supporting information


**Fig. S1** Linkage disequilibrium (LD) patterns.

**Table S1** Basic characteristics of the genotyped participants of each Framingham cohort.

**Table S2** The associations of genetic variants with lipids in each Framingham cohort.

**Table S3** The effect of lipid‐lowering treatment on the associations of genetic variants with total cholesterol.

**Table S4** The effect of lipid‐lowering treatment on the associations of genetic variants with high‐density lipoprotein cholesterol.

**Table S5** The effect of total cholesterol on the associations of rs693 and rs562338 with high‐density lipoprotein cholesterol.

**Table S6** The associations of rs693 and rs562338 with risks of myocardial infarction in each Framingham cohort.

**Table S7** Comparison of the associations of rs693 and rs562338 with risks for all onsets of myocardial infarction (MI) and for the prospective onsets only.

**Table S8** The effect of lipids on the pooled associations of rs693 and rs562338 with risks of myocardial infarction.

**Table S9** The effect of lipid‐lowering treatment on the associations of rs693 and rs562338 with risks of myocardial infarction.

**Table S10** The associations of rs693 and rs562338 with risks of overall death: the role of lipids and myocardial infarction (MI).

**Table S11** Pooled associations of rs693 and rs562338 with risks of mortality for MI patients.

**File S1** Acknowledgements statement.
