# Supplementary material for: Uncoupling associations of risk alleles with endophenotypes and phenotypes: insights from the ApoB locus and heart‐related traits
Source: Aging Cell. 2016 Sep 28;16(1):61–72. doi: 10.1111/acel.12526 (PMC5242299; doi:10.1111/acel.12526)
Supplement: Supplementary file 1 — Fig. S1 Linkage disequilibrium (LD) patterns. [file ACEL-16-61-s001.pdf]

Figure S1. Linkage disequilibrium (LD) patterns.

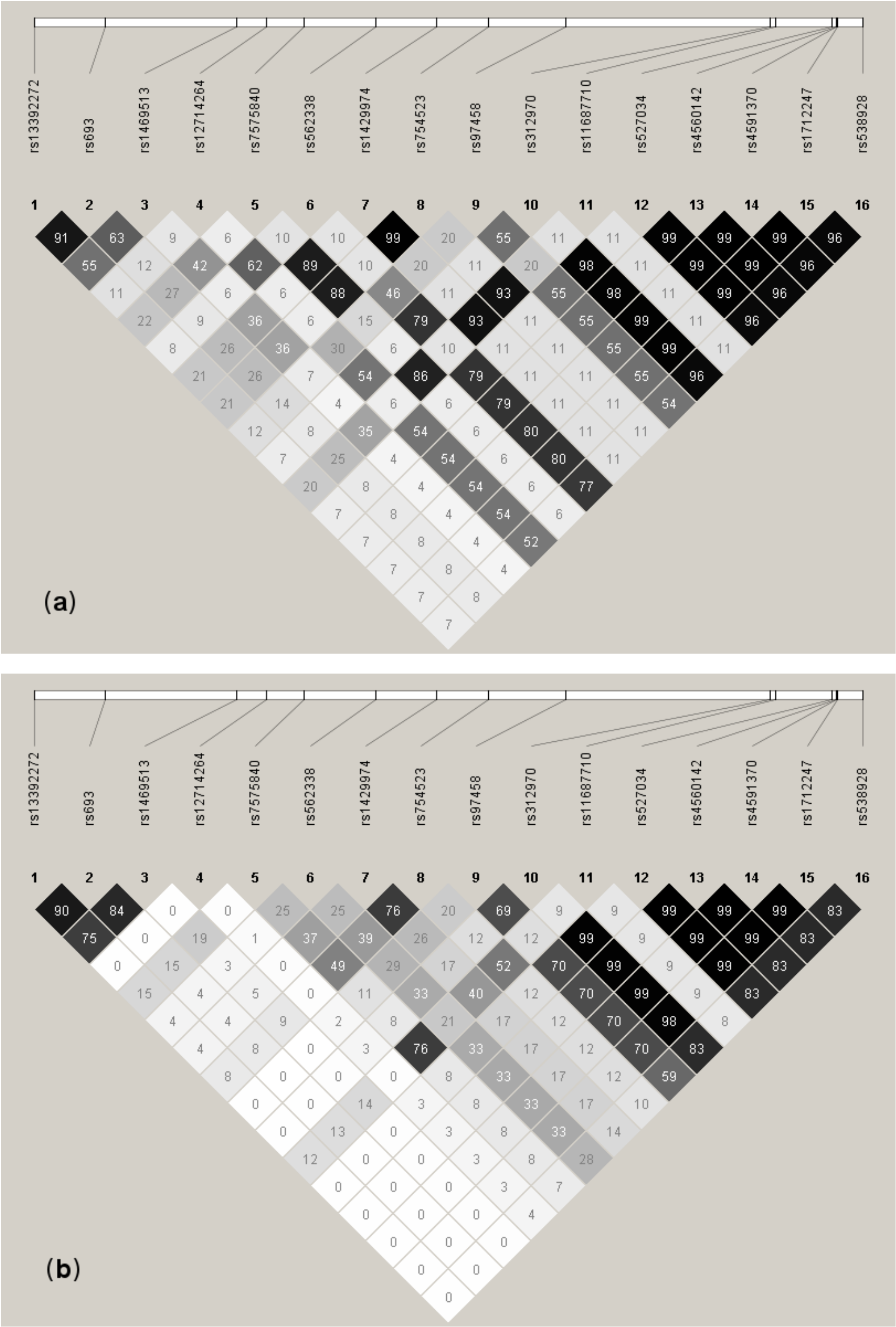

The LD patterns are for selected SNPs in the *ApoB* locus for (a) whites and (b) blacks in the ARIC study.

These LD patterns are representative of those for whites and blacks observed in the other studies.
