# Supplementary material for: Uncoupling associations of risk alleles with endophenotypes and phenotypes: insights from the ApoB locus and heart‐related traits
Source: Aging Cell. 2016 Sep 28;16(1):61–72. doi: 10.1111/acel.12526 (PMC5242299; doi:10.1111/acel.12526)
Supplement: Supplementary file 2 — Table S1 Basic characteristics of the genotyped participants of each Framingham cohort. [file ACEL-16-61-s002.pdf]

Table S1. Basic characteristics of the genotyped participants of each Framingham cohort.

Factor	rs693			rs562338		
	GG	GA	AA	GG	GA	AA
**Framingham Heart Study (FHS) original cohort**						
N (%[*])	270 (24.4)	570 (51.6)	265 (24)	699 (64.4)	341 (31.4)	45 (4.1)
Age, mean (SD), years	37.2 (5.8)	37.8 (6.1)	38.2 (5.9)	37.5 (5.8)	37.8 (6.2)	38 (5.5)
LS, mean (SD), years	88.1 (6.9)	88.4 (7)	88.1 (7.1)	88.3 (7)	88.4 (6.7)	87.8 (7.4)
TC, mean (SD), mg/dl[***]	233.7 (40.5)	238 (40.5)	244.8 (52.9)	240.6 (45.3)	236.2 (40.8)	221.7 (36.3)
HDL-C, mean (SD), mg/dl[***]	49 (12.8)	49.9 (14.7)	49.8 (15.3)	49.9 (14.5)	49.7 (14.8)	47.8 (11.1)
MI, yes (%[**])	79 (29.3)	148 (26)	56 (21.1)	174 (24.9)	88 (25.8)	11 (24.4)
Death, yes (%[**])	240 (88.9)	508 (89.1)	247 (93.2)	622 (89)	310 (90.9)	43 (95.6)
**FHS Offspring cohort**						
N (%[*])	953 (26.5)	1686 (46.9)	955 (26.6)	2320 (64.6)	1109 (30.9)	162 (4.5)
Age, mean (SD), years	35.1 (9.9)	35.3 (10)	35.6 (10.1)	35.4 (10.1)	35.2 (9.7)	35.1 (9.7)
LS, mean (SD), years	71.9 (9.4)	71.9 (9.1)	72.2 (9.3)	72 (9.3)	72 (9.1)	72.1 (9)
TC, mean (SD), mg/dl	189.7 (37.1)	193.6 (37.4)	198.8 (39.7)	196 (38.1)	190.4 (38)	187.8 (36.2)
HDL-C, mean (SD), mg/dl	52.3 (15.2)	50.9 (14.4)	50 (14.4)	50.7 (14.6)	51.6 (14.5)	51.5 (15.7)
MI, yes (%[**])	104 (10.9)	173 (10.3)	90 (9.4)	240 (10.3)	103 (9.3)	24 (14.8)
Death, yes (%[**])	182 (19.1)	363 (21.5)	202 (21.2)	502 (21.6)	217 (19.6)	26 (16)
**FHS 3rd Generation cohort**						
N (%[*])	1009 (26.7)	1789 (47.4)	978 (25.9)	2384 (63.2)	1195 (31.7)	194 (5.1)
Age, mean (SD), years	40.4 (8.8)	40.1 (8.9)	39.7 (8.9)	40 (9.1)	40.2 (8.5)	40.2 (8.8)
LS, mean (SD), years	48 (9)	47.8 (9.1)	47.4 (9.1)	47.6 (9.2)	47.9 (8.6)	47.6 (9.2)
TC, mean (SD), mg/dl	185.9 (38.3)	189.3 (34.4)	191.4 (34.5)	190.5 (35.8)	186.7 (35.2)	184.1 (33.4)
HDL-C, mean (SD), mg/dl	54.2 (15.6)	55 (16)	53.6 (16.7)	54.4 (16.1)	54.7 (16.2)	52.7 (14.9)
MI, yes (%[**])	7 (0.7)	16 (0.9)	16 (1.6)	22 (0.9)	15 (1.3)	2 (1)
Death, yes (%[**])	11 (1.1)	14 (0.8)	11 (1.1)	25 (1)	9 (0.8)	2 (1)

[*] Percentage is within the genotyped sample; [**] percentage is within each genotype.
N denotes sample size.
Mean age is given at baselines.
LS denotes lifespan, i.e., age at death or the end of follow-up.
TC denotes total cholesterol; HDL-C denotes high-density lipoprotein cholesterol; MI denotes myocardial infarction.
[***] Mean TC and HDL-C were measured at examination 9 for FHS original cohort because this examination included the largest sample size with non-missing information on these lipids. For the other FHS cohorts mean TC and HDL-C were representatively given at baselines.
