# Supplementary material for: Uncoupling associations of risk alleles with endophenotypes and phenotypes: insights from the ApoB locus and heart‐related traits
Source: Aging Cell. 2016 Sep 28;16(1):61–72. doi: 10.1111/acel.12526 (PMC5242299; doi:10.1111/acel.12526)
Supplement: Supplementary file 3 — Table S2 The associations of genetic variants with lipids in each Framingham cohort. [file ACEL-16-61-s003.pdf]

Table S2. The associations of genetic variants with lipids in each Framingham cohort.

Genetic factor, model	FHS Original Cohort, N=1,077			FHS Offspring, N=3,589			FHS 3rd Generation, N=3,770		
	Beta	SE	p-value	Beta	SE	p-value	Beta	SE	p-value
Total cholesterol (TC)									
rs693, M1	0.23	0.28	4.1E-01	0.88	0.14	6.9E-10	0.82	0.18	3.5E-06
rs562338, M1	-0.66	0.34	5.4E-02	-1.12	0.18	5.7E-10	-0.93	0.22	1.9E-05
rs693, M2	0.05	0.30	8.5E-01	0.64	0.15	3.0E-05	0.62	0.19	1.3E-03
rs562338, M2	-0.63	0.36	8.1E-02	-0.82	0.20	2.5E-05	-0.63	0.24	7.7E-03
PS-AA	-0.18	0.27	4.9E-01	0.18	0.14	2.1E-01	0.19	0.18	2.8E-01
PS-GA	-0.31	0.20	1.2E-01	-0.78	0.10	3.4E-14	-0.68	0.13	5.7E-08
High-density lipoprotein cholesterol (HDL-C)									
rs693, M1	-0.59	0.44	1.8E-01	-0.54	0.24	2.3E-02	-0.24	0.26	2.1E-01
rs562338, M1	-0.31	0.55	5.7E-01	0.08	0.30	7.9E-01	-0.18	0.32	3.1E-01
rs693, M2	-0.77	0.47	1.0E-01	-0.60	0.26	1.9E-02	-0.35	0.28	2.1E-01
rs562338, M2	-0.63	0.58	2.7E-01	-0.20	0.33	5.4E-01	-0.35	0.34	3.1E-01
PS-AA	-0.73	0.42	8.5E-02	-0.48	0.24	4.0E-02	-0.35	0.26	1.7E-01
PS-GA	0.29	0.32	3.5E-01	0.31	0.17	6.8E-02	0.12	0.18	5.2E-01

N denotes maximal number of individuals in the analyses with missing information on rs693, rs562338, total cholesterol, and high-density lipoprotein cholesterol excluded.
M1 denotes model 1 with one reference SNP included.
M2 denotes model 2 with both reference SNPs included.
PS-AA and PS-GA denote polygenic scores constructed as counts of the rs693_A or rs562338_A and rs693_G or rs562338_A alleles, respectively.
The effect size beta evaluated for $100\times\log_{10}$(TC) and $100\times\log_{10}$(HDL-C) is the estimate of cumulative genetic effects over multiple examinations using mixed effects regression model. Sign of beta indicates direction of the effect in additive genetic models. The effect alleles were: (i) allele(s) A in each SNP and in polygenic score PS-AA and (ii) alleles G and A in polygenic score PS-GA.
SE denotes standard error.
