# Supplementary material for: Uncoupling associations of risk alleles with endophenotypes and phenotypes: insights from the ApoB locus and heart‐related traits
Source: Aging Cell. 2016 Sep 28;16(1):61–72. doi: 10.1111/acel.12526 (PMC5242299; doi:10.1111/acel.12526)
Supplement: Supplementary file 4 — Table S3 The effect of lipid‐lowering treatment on the associations of genetic variants with total cholesterol. [file ACEL-16-61-s004.pdf]

Table S3. The effect of lipid lowering treatment on the associations of genetic variants with total cholesterol.

Genetic factor, model	ARIC, N=9,501			FHS, N=7,357			MESA, N=2,522			CHS, N=4,208		
	Beta	SE	p-value	Beta	SE	p-value	Beta	SE	p-value	Beta	SE	p-value
No adjustment for lipid lowering treatment												
rs693, M1	0.79	0.10	4.6E-15	0.88	0.12	2.9E-14	0.42	0.19	2.6E-02	0.61	0.16	1.0E-04
rs562338, M1	-1.14	0.13	9.4E-18	-1.07	0.15	1.4E-13	-0.76	0.23	1.0E-03	-1.18	0.20	3.3E-09
rs693, M2	0.58	0.11	3.6E-08	0.65	0.13	1.9E-07	0.25	0.20	2.1E-01	0.34	0.17	4.2E-02
rs562338, M2	-0.91	0.14	6.9E-11	-0.77	0.16	9.3E-07	-0.66	0.24	6.4E-03	-1.04	0.21	9.8E-07
PS-GA	-0.74	0.07	9.6E-24	-0.77	0.08	2.8E-20	-0.42	0.13	1.6E-03	-0.68	0.11	2.1E-09
Adjusted for lipid lowering treatment												
rs693, M1	0.83	0.10	1.8E-15	0.94	0.12	1.0E-14	0.55	0.19	2.8E-03	0.61	0.16	1.1E-04
rs562338, M1	-1.19	0.13	2.9E-18	-1.15	0.15	3.8E-14	-1.01	0.23	9.3E-06	-1.18	0.20	4.8E-09
rs693, M2	0.61	0.11	2.3E-08	0.69	0.13	1.3E-07	0.33	0.20	9.6E-02	0.34	0.17	4.2E-02
rs562338, M2	-0.95	0.14	3.4E-11	-0.82	0.13	4.8E-07	-0.88	0.24	2.4E-04	-1.03	0.21	1.3E-06
PS-GA	-0.78	0.08	1.4E-24	-0.82	0.09	5.1E-21	-0.56	0.13	2.3E-05	-0.68	0.11	2.6E-09

N denotes maximal number of individuals in the analyses with missing information on rs693, rs562338, total cholesterol, high-density lipoprotein cholesterol, and lipid lowering treatment excluded.
M1 denotes model 1 with one reference SNP included.
M2 denotes model 2 with both reference SNPs included.
PS-GA denotes polygenic scores constructed as counts of rs693_G or rs562338_A alleles.
The effect size beta evaluated for $100\times\log_{10}(TC)$ is the estimate of cumulative genetic effects over multiple examinations using mixed effects regression model. Sign of beta indicates direction of the effect in additive genetic models. The effect alleles were: (i) allele(s) A in each SNP and (ii) alleles G and A in polygenic score PS-GA.
SE denotes standard error.
