# Supplementary material for: Uncoupling associations of risk alleles with endophenotypes and phenotypes: insights from the ApoB locus and heart‐related traits
Source: Aging Cell. 2016 Sep 28;16(1):61–72. doi: 10.1111/acel.12526 (PMC5242299; doi:10.1111/acel.12526)
Supplement: Supplementary file 5 — Table S4 The effect of lipid‐lowering treatment on the associations of genetic variants with high‐density lipoprotein cholesterol. [file ACEL-16-61-s005.pdf]

Table S4. The effect of lipid lowering treatment on the associations of genetic variants with high-density lipoprotein cholesterol.

Genetic factor, model	ARIC, N=9,501			FHS, N=7,357			MESA, N=2,522			CHS, N=4,208		
	Beta	SE	p-value	Beta	SE	p-value	Beta	SE	p-value	Beta	SE	p-value
No adjustment for lipid lowering treatment												
rs693, M1	-0.31	0.17	6.6E-02	-0.36	0.18	4.1E-02	-0.13	0.30	6.7E-01	0.16	0.23	5.0E-01
rs562338, M1	0.02	0.22	9.4E-01	-0.12	0.22	6.0E-01	-0.19	0.37	6.1E-01	0.10	0.29	7.5E-01
rs693, M2	-0.34	0.18	5.7E-02	-0.46	0.19	1.6E-02	-0.20	0.32	5.4E-01	0.20	0.21	3.2E-01
rs562338, M2	-0.12	0.23	6.1E-01	-0.32	0.24	1.6E-01	-0.26	0.39	5.0E-01	0.18	0.31	5.6E-01
PS-AA	-0.28	0.16	8.8E-02	-0.43	0.18	1.5E-02	-0.22	0.28	4.5E-01	0.18	0.22	4.1E-01
Adjusted for lipid lowering treatment												
rs693, M1	-0.31	0.17	5.9E-02	-0.36	0.18	4.1E-02	-0.14	0.30	6.5E-01	0.16	0.23	5.0E-01
rs562338, M1	0.03	0.22	8.9E-01	-0.12	0.22	6.0E-01	-0.17	0.37	6.5E-01	0.09	0.29	7.5E-01
rs693, M2	-0.34	0.18	5.3E-02	-0.46	0.19	1.6E-02	-0.20	0.32	5.3E-01	0.20	0.24	4.0E-01
rs562338, M2	-0.11	0.23	6.5E-01	-0.33	0.24	1.6E-01	-0.25	0.39	5.3E-01	0.18	0.31	5.6E-01
PS-AA	-0.27	0.16	8.8E-02	-0.43	0.18	1.5E-02	-0.21	0.28	4.5E-01	0.18	0.22	4.0E-01

N denotes maximal number of individuals in the analyses with missing information on rs693, rs562338, total cholesterol, high-density lipoprotein cholesterol, and lipid lowering treatment excluded.
M1 denotes model 1 with one reference SNP included.
M2 denotes model 2 with both reference SNPs included.
PS-AA denotes polygenic scores constructed as counts of rs693_A or rs562338_A alleles.
The effect size beta evaluated for $100\times\log_{10}$(HDL-C) is the estimate of cumulative genetic effects over multiple examinations using mixed effects regression model. Sign of beta indicates direction of the effect in additive genetic models. The effect alleles were allele(s) A in each SNP and polygenic score PS-AA.
SE denotes standard error.
