# Supplementary material for: Uncoupling associations of risk alleles with endophenotypes and phenotypes: insights from the ApoB locus and heart‐related traits
Source: Aging Cell. 2016 Sep 28;16(1):61–72. doi: 10.1111/acel.12526 (PMC5242299; doi:10.1111/acel.12526)
Supplement: Supplementary file 6 — Table S5 The effect of total cholesterol on the associations of rs693 and rs562338 with high‐density lipoprotein cholesterol. [file ACEL-16-61-s006.pdf]

Table S5. The effect of total cholesterol on the associations of rs693 and rs562338 with high-density lipoprotein cholesterol.

Genetic factor, model	ARIC, N=9,585			FHS, N=8,436			MESA, N=2,680			CHS, N=4,208		
	Beta	SE	p-value	Beta	SE	p-value	Beta	SE	p-value	Beta	SE	p-value
No adjustment for total cholesterol												
rs693, M1	-0.32	0.17	5.6E-2	-0.41	0.17	1.5E-2	-0.04	0.29	8.9E-1	0.16	0.23	5.0E-1
rs562338, M1	0.04	0.22	8.4E-1	-0.16	0.21	4.5E-1	-0.19	0.36	5.9E-1	0.10	0.29	7.5E-1
rs693, M2	-0.34	0.18	5.3E-2	-0.52	0.18	3.5E-3	-0.10	0.31	7.4E-1	0.20	0.21	3.2E-1
rs562338, M2	-0.09	0.23	7.0E-1	-0.39	0.22	7.6E-2	-0.23	0.38	5.4E-1	0.18	0.31	5.6E-1
Adjusted for total cholesterol												
rs693, M1	-0.39	0.17	2.1E-02	-0.50	0.17	2.8E-03	-0.09	0.29	7.5E-01	0.04	0.23	8.6E-01
rs562338, M1	0.14	0.22	5.2E-01	-0.04	0.21	8.6E-01	-0.08	0.36	8.2E-01	0.31	0.29	2.7E-01
rs693, M2	-0.39	0.18	2.7E-02	-0.59	0.18	1.0E-03	-0.13	0.31	6.7E-01	0.14	0.24	5.7E-01
rs562338, M2	-0.01	0.23	9.5E-01	-0.30	0.22	1.7E-01	-0.13	0.38	7.2E-01	0.38	0.31	2.2E-01

N denotes maximal number of individuals in the analyses with missing information on rs693, rs562338, total cholesterol, and high-density lipoprotein cholesterol excluded.

M1 denotes model 1 with one reference SNP included.

M2 denotes model 2 with both reference SNPs included.

The effect size beta evaluated for $100\times\log_{10}$(HDL-C) is the estimate of cumulative genetic effects over multiple examinations using mixed effects regression model. Sign of beta indicates direction of the effect in additive genetic models with allele A considered as an effect allele in each SNP.

SE denotes standard error.
