# Supplementary material for: Uncoupling associations of risk alleles with endophenotypes and phenotypes: insights from the ApoB locus and heart‐related traits
Source: Aging Cell. 2016 Sep 28;16(1):61–72. doi: 10.1111/acel.12526 (PMC5242299; doi:10.1111/acel.12526)
Supplement: Supplementary file 7 — Table S6 The associations of rs693 and rs562338 with risks of myocardial infarction in each Framingham cohort. [file ACEL-16-61-s007.pdf]

Table S6. The associations of rs693 and rs562338 with risks of myocardial infarction in each Framingham cohort.

Genetic factor, model	FHS, $N_{T/C}$=981/238			FHSO, $N_{T/C}$=3504/349			3rd Gen, $N_{T/C}$=3726/19		
	Beta	SE	p-value	Beta	SE	p-value	Beta	SE	p-value
rs693, M1	-0.25	0.09	8.2E-03	-0.14	0.07	6.0E-02	0.36	0.32	2.7E-01
rs562338, M1	0.02	0.12	8.6E-01	0.07	0.09	4.8E-01	0.26	0.36	4.7E-01
rs693, M2	-0.27	0.10	6.0E-03	-0.14	0.08	8.1E-02	0.55	0.36	1.3E-01
rs562338, M2	-0.09	0.12	4.6E-01	0.01	0.10	9.6E-01	0.54	0.41	1.9E-01

$N_{T/C}$ denotes total number (T) of individuals in the analyses and the number of cases (C) among them.
M1 denotes model 1 with one reference SNP included.
M2 denotes model 2 with both reference SNPs included.
The effect beta was evaluated in the Cox proportional hazard regression model. Sign of beta indicates direction of the effect in additive genetic models with alleles A considered as effect alleles for each SNP.
SE denotes standard error.
