# Supplementary material for: Uncoupling associations of risk alleles with endophenotypes and phenotypes: insights from the ApoB locus and heart‐related traits
Source: Aging Cell. 2016 Sep 28;16(1):61–72. doi: 10.1111/acel.12526 (PMC5242299; doi:10.1111/acel.12526)
Supplement: Supplementary file 8 — Table S7 Comparison of the associations of rs693 and rs562338 with risks for all onsets of myocardial infarction (MI) and for the prospective onsets only. [file ACEL-16-61-s008.pdf]

Table S7. Comparison of the associations of rs693 and rs562338 with risks for all onsets of myocardial infarction (MI) and for the prospective onsets only.

Genetic factor, model	FHS^{*}_{all}, $N_{T/C}$=4,485/587			FHS^{*}_{prsp}, $N_{T/C}$=4,186/289			CHS_{all}, $N_{T/C}$=4,196/931			CHS_{prsp}, $N_{T/C}$=4,147/884		
	Beta	SE	p-value	Beta	SE	p-value	Beta	SE	p-value	Beta	SE	p-value
rs693, M1	-0.18	0.06	1.8E-03	-0.22	0.08	8.7E-03	0.13	0.05	6.8E-03	0.12	0.05	1.7E-02
rs562338, M1	0.04	0.07	5.8E-01	0.11	0.10	2.6E-01	-0.02	0.06	8.0E-01	-0.02	0.06	7.7E-01
rs693, M2	-0.19	0.06	1.7E-03	-0.21	0.09	1.7E-02	0.14	0.05	5.6E-03	0.13	0.05	1.5E-02
rs562338, M2	-0.04	0.08	5.8E-01	0.02	0.11	8.3E-01	0.04	0.06	5.0E-01	0.04	0.06	6.1E-01

$N_{T/C}$ denotes total number (T) of individuals in the analyses and the number of cases (C) among them.
M1 denotes model 1 with one reference SNP included.
M2 denotes model 2 with both reference SNPs included.
The effect beta was evaluated in the Cox proportional hazard regression model. Sign of beta indicates direction of the effect in additive genetic models with alleles A considered as effect alleles for each SNP.
SE denotes standard error.
[*] The 3rd generation cohort of the Framingham Heart Study (FHS) was not included because of small number of MI events (N=19).
Subscript “all” denotes retrospective and prospective onsets of MI.
Subscript “prsp” denotes only prospective onsets of MI.
