# Supplementary material for: Uncoupling associations of risk alleles with endophenotypes and phenotypes: insights from the ApoB locus and heart‐related traits
Source: Aging Cell. 2016 Sep 28;16(1):61–72. doi: 10.1111/acel.12526 (PMC5242299; doi:10.1111/acel.12526)
Supplement: Supplementary file 9 — Table S8 The effect of lipids on the pooled associations of rs693 and rs562338 with risks of myocardial infarction. [file ACEL-16-61-s009.pdf]

Table S8. The effect of lipids on the pooled associations of rs693 and rs562338 with risks of myocardial infarction.

Genetic factor, model	Lipid adjustment	All $N_{T/C}$=20,748/2,357			ARIC + FHS* $N_{T/C}$=14,047/1,351			MESA + CHS $N_{T/C}$=6,701/1,006			Absolute $N_{T/C}$=20,748/2,357		
		Beta	SE	p-value	Beta	SE	p-value	Beta	SE	p-value	Beta	SE	p-value
rs693, M1	No	-0.01	0.03	6.7E-1	-0.12	0.04	2.0E-3	0.13	0.05	3.7E-3	0.13	0.03	1.5E-5
rs562338,M1	No	-0.02	0.04	6.2E-1	-0.02	0.05	6.4E-1	-0.01	0.06	8.1E-1	0.04	0.04	2.5E-1
rs693, M2	No	-0.02	0.03	5.3E-1	-0.14	0.04	5.9E-4	0.15	0.05	2.8E-3	0.15	0.03	3.3E-6
rs562338, M2	No	-0.03	0.04	5.0E-1	-0.08	0.05	1.2E-1	0.05	0.06	4.4E-1	0.07	0.04	7.9E-2
rs693, M1	TC	-0.03	0.03	2.8E-1	-0.16	0.04	7.2E-5	0.13	0.05	4.2E-3	0.15	0.03	5.5E-7
rs562338, M1	TC	0.006	0.04	8.8E-1	0.02	0.05	6.7E-1	-0.01	0.06	8.5E-1	0.05	0.04	2.2E-1
rs693, M2	TC	-0.03	0.03	2.7E-1	-0.17	0.04	5.0E-5	0.14	0.05	3.1E-3	0.16	0.03	2.9E-7
rs562338, M2	TC	-0.01	0.04	8.3E-1	-0.05	0.05	3.7E-1	0.05	0.06	4.3E-1	0.05	0.04	2.0E-1
rs693, M1	TC+HDL-C	-0.04	0.03	1.6E-1	-0.17	0.04	2.0E-5	0.13	0.05	4.9E-3	0.16	0.03	1.8E-7
rs562338, M1	TC+HDL-C	0.01	0.04	8.0E-1	0.02	0.05	7.4E-1	0.004	0.06	9.5E-1	0.04	0.04	2.5E-1
rs693, M2	TC+HDL-C	-0.04	0.03	1.6E-1	-0.18	0.04	1.1E-5	0.15	0.05	2.6E-3	0.17	0.03	5.5E-8
rs562338, M2	TC+HDL-C	-0.01	0.04	8.3E-1	-0.06	0.05	2.8E-1	0.07	0.06	2.8E-1	0.06	0.04	1.2E-1

$N_{T/C}$ denotes total number (T) of individuals in the analyses and the number of cases (C) among them.
M1 denotes model 1 with one reference SNP included.
M2 denotes model 2 with both reference SNPs included.
"No" denotes no adjustment for lipids (total cholesterol [TC] and high-density lipoprotein cholesterol [HDL-C]); "TC" denotes adjustment for TC, and "TC+HDL-C" denotes adjustment for TC and HDL-C.
The effect beta was evaluated in the Cox proportional hazard regression model. Sign of beta indicates direction of the effect in additive genetic models with alleles A considered as effect alleles for each SNP.
SE denotes standard error.
Columns "All" show the results of mega-analysis of all studies, which resembled conventional strategy of meta-analyses in GWAS pooling the estimated effects from each study.
Columns "ARIC + FHS*" and "MESA + CHS" show the results of mega-analyses of more homogeneous samples.
Columns "Absolute" show the results of the meta-analysis pooling the associations from all studies regardless of the effect directions, i.e., for absolute values of the effects. Meta-analysis was conducted because the proportionality assumption in the Cox proportional hazard model was violated in this case.
* The 3rd generation cohort of the Framingham Heart Study (FHS) was not included because of small number of events (N=19).
