# Supplementary material for: Uncoupling associations of risk alleles with endophenotypes and phenotypes: insights from the ApoB locus and heart‐related traits
Source: Aging Cell. 2016 Sep 28;16(1):61–72. doi: 10.1111/acel.12526 (PMC5242299; doi:10.1111/acel.12526)
Supplement: Supplementary file 10 — Table S9 The effect of lipid‐lowering treatment on the associations of rs693 and rs562338 with risks of myocardial infarction. [file ACEL-16-61-s010.pdf]

Table S9. The effect of lipid lowering treatment on the associations of rs693 and rs562338 with risks of myocardial infarction.

Genetic factor, model	ARIC, N=9,430/749			FHS*, N=3,490/349			MESA, N=2,504/75			CHS, N=4,193/930		
	Beta	SE	p-value	Beta	SE	p-value	Beta	SE	p-value	Beta	SE	p-value
No adjustment for lipid lowering treatment												
rs693, M1	-0.12	0.05	2.4E-2	-0.23	0.08	2.6E-3	0.16	0.17	3.4E-1	0.13	0.05	6.9E-3
rs562338, M1	-0.04	0.07	5.5E-1	0.14	0.09	1.4E-1	0.02	0.21	9.3E-1	-0.00	0.06	1.0E-0
rs693, M2	-0.14	0.06	1.0E-2	-0.21	0.08	7.4E-3	0.19	0.18	3.0E-1	0.15	0.05	4.2E-3
rs562338, M2	-0.10	0.07	1.8E-1	0.04	0.10	6.8E-1	0.09	0.22	6.7E-1	0.06	0.07	3.3E-1
Adjusted for lipid lowering treatment												
rs693, M1	-0.12	0.05	2.5E-2	-0.23	0.07	2.0E-3	0.16	0.17	3.3E-1	0.13	0.05	6.3E-3
rs562338, M1	-0.04	0.07	5.5E-1	0.15	0.09	1.2E-1	0.02	0.21	9.4E-1	-0.00	0.06	1.0E-0
rs693, M2	-0.14	0.06	1.1E-2	-0.22	0.08	6.4E-3	0.19	0.17	2.9E-1	0.15	0.05	3.8E-3
rs562338, M2	-0.10	0.07	1.8E-1	0.05	0.10	6.2E-1	0.09	0.22	6.8E-1	0.06	0.07	3.3E-1

$N_{T/C}$ denotes total number (T) of individuals in the analyses and the number of cases (C) among them.
M1 denotes model 1 with one reference SNP included.
M2 denotes model 2 with both reference SNPs included.
The effect beta was evaluated in the Cox proportional hazard regression model. Sign of beta indicates direction of the effect in additive genetic models with alleles A considered as effect alleles for each SNP.
SE denotes standard error.
The models were adjusted for basic covariates, total cholesterol, and high-density lipoprotein cholesterol.
* The 3rd generation cohort of the Framingham Heart Study (FHS) was not included because of small number of events (N=19).
