# Supplementary material for: Uncoupling associations of risk alleles with endophenotypes and phenotypes: insights from the ApoB locus and heart‐related traits
Source: Aging Cell. 2016 Sep 28;16(1):61–72. doi: 10.1111/acel.12526 (PMC5242299; doi:10.1111/acel.12526)
Supplement: Supplementary file 11 — Table S10 The associations of rs693 and rs562338 with risks of overall death: the role of lipids and myocardial infarction (MI). [file ACEL-16-61-s011.pdf]

Table S10. The associations of rs693 and rs562338 with risks of overall death: the role of lipids and myocardial infarction (MI).

SNPs	ARIC, $N_{T/C}$=9,407/1,372			FHS*, $N_{T/C}$=4,409/1,591			MESA, $N_{T/C}$=2,475/174			CHS, $N_{T/C}$=4,196/2,131		
	Beta	SE	p-value	Beta*	SE	p-value	Beta*	SE	p-value	Beta*	SE	p-value
No adjustment for lipids and MI												
rs693, M1	-0.06	0.04	1.1E-01	-0.01	0.04	7.8E-01	-0.24	0.11	3.1E-02	0.06	0.03	6.7E-02
rs562338, M1	0.01	0.05	7.8E-01	-0.03	0.05	5.0E-01	0.26	0.13	4.2E-02	-0.006	0.04	8.9E-01
rs693, M2	-0.07	0.04	1.1E-01	-0.02	0.04	5.9E-01	-0.19	0.12	1.1E-01	0.07	0.03	4.0E-02
rs562338, M2	-0.01	0.05	8.2E-01	-0.04	0.05	4.2E-01	0.19	0.13	1.7E-01	0.02	0.04	6.0E-01
Adjusted for lipids												
rs693, M1	-0.08	0.04	5.5E-02	-0.03	0.04	5.0E-01	-0.23	0.11	3.8E-02	0.07	0.03	4.0E-02
rs562338, M1	0.02	0.05	7.6E-01	-0.01	0.05	7.9E-01	0.25	0.13	4.5E-02	-0.006	0.04	8.8E-01
rs693, M2	-0.08	0.04	5.6E-02	-0.03	0.04	4.2E-01	-0.18	0.12	1.2E-01	0.07	0.03	3.4E-02
rs562338, M2	-0.02	0.05	7.7E-01	-0.03	0.05	6.0E-01	0.19	0.13	1.6E-01	0.02	0.04	5.9E-01
Adjusted for lipids and MI												
rs693, M1	-0.07	0.04	7.4E-02	0.00	0.04	1.0E-00	-0.24	0.11	3.9E-02	0.05	0.03	7.7E-02
rs562338, M1	0.02	0.05	7.4E-01	-0.02	0.05	6.7E-01	0.26	0.13	4.7E-02	-0.003	0.04	9.3E-01
rs693, M2	-0.07	0.04	7.7E-02	-0.01	0.04	8.8E-01	-0.18	0.12	1.2E-01	0.06	0.03	6.7E-02
rs562338, M2	-0.01	0.05	8.2E-01	-0.02	0.05	6.5E-01	0.19	0.14	1.4E-01	0.01	0.04	6.1E-01

$N_{T/C}$ denotes total number (T) of individuals in the analyses and the number of cases (C) among them.
M1 denotes model 1 with one reference SNP included.
M2 denotes model 2 with both reference SNPs included.
Lipids include total cholesterol and high-density lipoprotein cholesterol.
The effect beta was evaluated in the Cox proportional hazard regression model. Sign of beta indicates direction of the effect in additive genetic models with alleles A considered as effect alleles for each SNP.
SE denotes standard error.
* The 3rd generation cohort of the Framingham Heart Study (FHS) was not included because of small number of events (N=19).
