# Supplementary material for: Uncoupling associations of risk alleles with endophenotypes and phenotypes: insights from the ApoB locus and heart‐related traits
Source: Aging Cell. 2016 Sep 28;16(1):61–72. doi: 10.1111/acel.12526 (PMC5242299; doi:10.1111/acel.12526)
Supplement: Supplementary file 12 — Table S11 Pooled associations of rs693 and rs562338 with risks of mortality for MI patients. [file ACEL-16-61-s012.pdf]

Table S11. Pooled associations of rs693 and rs562338 with risks of mortality for MI patients.

Genetic factor, model	All, N_C=1,267			ARIC+FHS*, N_C=611			MESA+CHS, N_C=656			Absolute, N_C=1,267		
	Beta	SE	p-value	Beta	SE	p-value	Beta	SE	p-value	Beta	SE	p-value
rs693, M21	-0.04	0.04	2.9E-1	-0.23	0.06	1.4E-4	0.13	0.06	2.9E-2	0.18	0.04	2.2E-5
rs562338, M21	-0.03	0.05	6.1E-1	-0.14	0.08	7.0E-2	0.08	0.08	2.7E-1	0.12	0.06	2.9E-2
rs693, M22	-0.04	0.04	3.1E-1	-0.24	0.06	8.3E-5	0.14	0.06	1.9E-2	0.18	0.04	3.5E-5
rs562338, M22	-0.03	0.05	5.7E-1	-0.15	0.08	5.9E-2	0.09	0.08	2.5E-1	0.13	0.06	1.5E-2

Columns "All" show the results of mega-analysis of all studies, which resembled conventional strategy of meta-analyses in GWAS pooling the estimated effects from each study.

Columns "ARIC + FHS*" and "MESA + CHS" show the results of mega-analyses of more homogeneous samples.

Columns "Absolute" show the results of the meta-analysis pooling the associations from all studies regardless of the effect directions, i.e., for absolute values of the effects. Meta-analysis was conducted because the proportionality assumption in the Cox proportional hazard model was violated in this case.

M21 denoted the model with cases defined as death among MI patients and the others were considered as controls. The model included both reference SNPs and was adjusted for basic covariates, TC, and HDL-C. The total samples were: All and Absolute, N_T=20,748; ARIC+FHS*, N_T=14,047; MESA+CHS, N_T=6,701.

M22 was the same as M21 except the controls were individuals with no MI. The total samples were: All and Absolute, N_T=19,658; ARIC+FHS*, N_T=13,307; MESA+CHS, N_T=6,351.

The number of cases (N_C) in the analyses is given in the table.

* The 3rd generation cohort of the Framingham Heart Study (FHS) was not included because of small number of deaths after myocardial infarction (MI) events (N=5).

SE denotes standard error.
