# Supplementary material for: Uncoupling associations of risk alleles with endophenotypes and phenotypes: insights from the ApoB locus and heart‐related traits
Source: Aging Cell. 2016 Sep 28;16(1):61–72. doi: 10.1111/acel.12526 (PMC5242299; doi:10.1111/acel.12526)
Supplement: Supplementary file 13 — File S1 Acknowledgements statement. [file ACEL-16-61-s013.pdf]

Acknowledgments

The research reported in this paper was supported by Grants No P01 AG043352 and R01AG047310 from the National Institute on Aging (NIA). The funders had no role in study design, data collection and analysis, decision to publish, or preparation of the manuscript. The content is solely the responsibility of the authors and does not necessarily represent the official views of the National Institutes of Health (NIH). This manuscript was prepared using a limited access datasets obtained though dbGaP (accession numbers phs000007.v22.p8, phs000280.v2.p1, phs000209.v12.p3, phs000287.v3.p1).

The Framingham Heart Study (FHS) is conducted and supported by the National Heart, Lung, and Blood Institute (NHLBI) in collaboration with Boston University (Contract No. N01-HC-25195). This manuscript was not prepared in collaboration with investigators of the FHS and does not necessarily reflect the opinions or views of the FHS, Boston University, or NHLBI. Funding for SHARe Affymetrix genotyping was provided by NHLBI Contract N02-HL-64278. SHARe Illumina genotyping was provided under an agreement between Illumina and Boston University.

The Atherosclerosis Risk in Communities Study (ARIC) is carried out as a collaborative study supported by the NHLBI contracts (HHSN268201100005C, HHSN268201100006C, HHSN268201100007C, HHSN268201100008C, HHSN268201100009C, HHSN268201100010C, HHSN268201100011C, and HHSN268201100012C). The authors thank the staff and participants of the ARIC study for their important contributions. Funding for GENEVA was provided by the National Human Genome Research Institute grant U01HG004402 (E. Boerwinkle).

Multi-Ethnic Study of Atherosclerosis (MESA) and the MESA SHARe project are conducted and supported by the NHLBI in collaboration with MESA investigators. Support for MESA is provided by contracts N01-HC-95159, N01-HC-95160, N01-HC-95161, N01-HC-95162, N01-HC-95163, N01-HC-95164, N01-HC-95165, N01-HC-95166, N01-HC-95167, N01-HC-95168, N01-HC-95169 and CTSA UL1-RR-024156. Funding for SHARe genotyping was provided by NHLBI Contract N02-HL-64278.

This manuscript was not prepared in collaboration with MESA investigators and does not necessarily reflect the opinions or views of MESA, or the NHLBI.

The Cardiovascular Health Study (CHS) was supported by contract numbers N01-HC- 85079, N01-HC-85080, N01-HC-85081, N01-HC-85082, N01-HC-85083, N01-HC- 85084, N01-HC-85085, N01-HC-85086, N01-HC-35129, N01 HC-15103, N01 HC- 55222, N01-HC-75150, N01-HC-45133, N01-HC-85239 and HHSN268201200036C; grant numbers U01 HL080295 from the NHLBI and R01 AG-023629 from the NIA, with additional contribution from the National Institute of Neurological Disorders and Stroke. A full list of principal CHS investigators and institutions can be found at http://www.chs-nhlbi.org/pi.htm. This manuscript was not prepared in collaboration with CHS investigators and does not necessarily reflect the opinions or views of CHS, or the NHLBI. Support for the genotyping through the CARe Study was provided by NHLBI Contract N01-HC-65226.
